# A New Questionnaire to Assess Respiratory Symptoms (The Respiratory Symptom Experience Scale): Quantitative Psychometric Assessment and Validation Study

**DOI:** 10.2196/44036

**Published:** 2023-04-14

**Authors:** Saul Shiffman, Stacey A McCaffrey, Michael J Hannon, Nicholas I Goldenson, Ryan A Black

**Affiliations:** 1 PinneyAssociates, Inc Pittsburgh, PA United States; 2 JUUL Labs, Inc Washington, DC United States

**Keywords:** measure development, respiratory symptoms, COPD, e-cigarettes, electronic nicotine delivery system, ENDS, smoking, respiratory disease, respiratory health, health intervention, questionnaire, validation, validate, development, respiratory, pulmonary, lung, smoker, smoking

## Abstract

**Background:**

Smokers often experience respiratory symptoms (eg, morning cough), and those who stop smoking, including those who do so by switching completely to electronic nicotine delivery systems (ENDS), may experience reductions in symptoms. Existing respiratory symptom questionnaires may not be suitable for studying these changes, as they are intended for patient populations, such as those with chronic obstructive pulmonary disease (COPD).

**Objective:**

This study aimed to develop a respiratory symptom questionnaire appropriate for current smokers and for assessing changes when smokers stop smoking.

**Methods:**

The Respiratory Symptom Experience Scale (RSES) was derived from existing instruments and subject matter expert input and refined through cognitive debriefing interviews (n=49). Next, for purposes of the quantitative psychometric evaluation, the RSES was administered to smokers (n=202), former smokers (no tobacco use in >6 months; n=200), and switchers (n=208, smokers who switched to ENDS for >6 months), all of whom had smoked for at least 10 years (mean age 33 years). Participants, who averaged 62 (SD 12) years of age, included 28% (173/610) with respiratory allergy symptoms and 17% (104/610) with COPD. Test-retest reliability was assessed by repeat assessment after 1 week in 128 participants.

**Results:**

A generalized partial credit model confirmed that the response options were ordered, and a parallel analysis using principal components confirmed that the scale was unidimensional. With allowance for 2 sets of correlated errors between pairs of items, a 1-factor graded response model fit the data. Discrimination parameters were approximately 1 or greater for all items. Scale reliability was 0.80 or higher across a broad range of severity (standardized scores −0.40 to 3.00). Test-retest reliability (absolute intraclass correlation) was good, at 0.89. RSES convergent validity was supported by substantial differences (Cohen *d*=0.74) between those with and without a diagnosis of respiratory disease (averaging 0.57 points, indicating that differences of this size or smaller represent meaningful differences). RSES scores also strongly differentiated those with and without COPD (*d*=1.52). Smokers’ RSES scores were significantly higher than former smokers’ scores (*P*<.001). Switchers’ RSES scores were significantly lower than smokers’ scores (*P*<.001) and no different from former smokers’ scores (*P*=.34).

**Conclusions:**

The RSES fills an important gap in the existing toolkit of respiratory symptom questionnaires; it is a reliable and valid tool to assess respiratory symptoms in adult current and former smokers, including those who have switched to noncombusted nicotine products. This suggests that the scale is sensitive to respiratory symptoms that develop in smokers and to their remission when smokers quit or switch to noncombusted nicotine products intended to reduce the harm of smoking. The findings also suggest that switching from cigarettes to ENDS may improve respiratory health.

## Introduction

Cigarette smoke contains thousands of chemicals that are inhaled and deposited in the lungs, causing inflammation of the airways, impairing ciliary clearance, and leading to oxidative injury [1-3]. Cigarette smoking is associated with chronic respiratory diseases (eg, chronic obstructive pulmonary disease [COPD]), a severe, late-developing inflammatory lung disease that causes airflow blockage and breathing-related problems [1-3]. Although respiratory diseases such as COPD are associated with particular pathophysiological developments and are reflected in objective methods such as spirometry, they are expressed in and often diagnosed by respiratory symptoms such as cough, wheezing, and sputum production [4]. However, smokers often experience such symptoms even without having (yet) developed diagnosable COPD [1-3].

Numerous validated self-report measures for assessing respiratory symptoms have been published. These questionnaires were primarily developed and validated specifically for use in patient populations who already had COPD. However, such measures may not be appropriate for assessing respiratory symptoms in those without a respiratory condition but who are experiencing respiratory symptoms. First, many of these questionnaires include items that assess severe disease states that may not be applicable to smokers without clinical respiratory disease, for example, “I am not at all confident leaving my home because of my lung condition” (COPD Assessment Test [5]), “I feel that I am not in control of my chest problems” (St. George’s Respiratory Symptom Questionnaire [6]), “In the past 2 weeks, my cough has interrupted conversation or telephone calls” (Leicester Cough Questionnaire [7]), and “on average, during the past week, how often did you feel depressed (down) because of your breathing problems?” (Clinical COPD Questionnaire [8], emphasis in the original). Second, when a questionnaire’s items are too “difficult” (ie, reflect symptoms of severe pulmonary disease) for the population being assessed, this leads to reduced measurement precision and can also result in a “floor effect,” inhibiting measurement of the true reduction in respiratory symptoms over time. Third, scoring and interpretation of existing measures developed and validated specifically for diseased populations cannot be assumed to extend to nondiseased populations. Thus, a respiratory symptom questionnaire appropriate for use with populations without pulmonary disease who may be experiencing mild-to-moderate pulmonary symptoms, such as adult current and former smokers, is needed.

Electronic nicotine delivery systems (ENDS) deliver nicotine without many of the toxic combustion products associated with smoking and are intended to offer adult smokers a lower-risk alternative to smoking. Reviews by national health authorities, such as the National Academies of Sciences, Engineering, and Medicine [9] and the Royal College of Physicians [10], conclude that ENDS reduce users’ exposure to toxicants and carcinogens compared with combustible cigarettes. Nevertheless, using ENDS involves inhaling an aerosol with nicotine and other molecules. The literature is mixed regarding the respiratory effects of ENDS; some reviews have concluded that switching from cigarette smoking to ENDS is associated with reduced levels of self-reported respiratory symptoms [11,12], whereas others have concluded that the existing evidence is not sufficient to reach that conclusion [13]. Contributing to the difficulty in addressing this research question is the lack of an appropriate self-report measure of respiratory symptoms. Accordingly, a valid self-report measure of respiratory symptoms appropriate for smokers—including those without diagnosed respiratory disease—is needed to determine whether switching completely from combustible cigarettes to ENDS leads to a reduction in respiratory symptoms.

Therefore, the primary objective of this research was to develop and validate a respiratory symptom questionnaire appropriate for a general population of adult current and former smokers, including those without COPD. The Respiratory Symptom Experience Scale (RSES) was initially developed from existing instruments, with expert input, and then refined through multiple waves of semistructured cognitive debriefing interviews. Finally, the RSES was administered to adult current cigarette smokers (“Smokers”), adult former smokers who switched completely from cigarettes to ENDS for >6 months (“Switchers”), and adult former smokers who had not used any tobacco product in >6 months (“Former Smokers”) for purposes of the psychometric evaluation. Instrument development and validation included anticipated end users of the questionnaire (ie, smokers and switchers without COPD) to ensure that the resulting questionnaire would be valid for use with these populations. Former smokers and participants who reported a diagnosis of COPD or another respiratory symptom–related disease (eg, asthma) were also included for purposes of the psychometric evaluation.

Data from the psychometric evaluation also provided an opportunity for exploratory analyses examining how respiratory symptoms reported by switchers compared with symptoms reported by smokers and former smokers.

## Methods

### Questionnaire Drafting and Cognitive Testing

The questionnaire was drafted in accordance with principles outlined by prominent research organizations (eg, ISPOR) and United States Food & Drug Administration guidance. Content for the initial RSES was generated based on the review of relevant literature, including existing respiratory symptom or cough questionnaires and national or international surveys. Feedback from subject matter experts, including 5 pulmonologists, was used to refine the draft questionnaire.

Next, the draft questionnaire was evaluated through individual semistructured cognitive debriefing interviews with a total of 49 smokers and switchers. Participants were diverse with respect to demographics and included individuals with low health literacy. Participant feedback was used to rectify potential sources of confusion or response error in the questionnaire, to modify item content to enhance relevance and meaningfulness to participants, and to provide evidence of content validity.

The RSES generated from cognitive testing is presented in Textbox 1. The RSES is written below an eighth grade reading level (Flesch-Kincaid Grade Level=7.7).

**Respiratory Symptom Experience Scale.**

**Instructions**

- For the following questions, please think about your experiences in the past 30 days

**Response options (the same set of response options are presented with each item. See the Administration and Scoring section)**

- Never (0 days out of the last 30 days)
- Rarely (1-5 days)
- Occasionally (6-15 days)
- Most days (16-29 days)
- Every day (all 30 days out of the last 30 days)

**Item 1: morning cough**

- Morning cough with phlegm or mucus

**Item 2: cough frequently**

- Cough frequently throughout the day

**Item 3: shortness of breath**

- My shortness of breath makes it difficult to do normal daily activities such as walking up a flight of stairs or carrying a heavy object

**Item 4: easily winded**

- Becoming easily winded during normal daily activities (eg, doing laundry and carrying groceries)

**Item 5: wheezing**

- Wheezing or whistling in your chest at times when you are not exercising or doing other physically strenuous daily activities (eg, while resting)

### Quantitative Psychometric Evaluation

#### Procedures

The RSES was administered in a web-based survey of roughly equal numbers of smokers, switchers, and former smokers recruited from opt-in web-based research panels managed by Kantar Profiles from February to March 2021. Panelists were compensated for their time with panel points.

The survey included the RSES as well as global health status [14] and diagnostic status (Has a doctor, nurse, or other health professional told you that you have any of the following? Choose all that apply. *Response options:* allergies, asthma, chronic bronchitis, COPD, emphysema, other lung or respiratory condition, obesity, congestive heart failure, none of the above, don’t know) items for purposes of evaluating the validity of the RSES.

It was expected that (1) RSES scores would be higher among those with COPD (ie, chronic bronchitis, emphysema, or COPD) or other respiratory symptom–related diseases, and that (2) RSES scores would be uniquely associated with a diagnosis of COPD above and beyond the other respiratory symptom–related diagnoses.

Approximately 1 week after completing this survey, participants were recontacted and asked to complete the RSES a second time (Time 2 survey) for purposes of evaluating test-retest reliability. Time 2 survey invitations stopped once the target sample size was reached. A 1-week interval was selected to make it unlikely that symptoms had actually changed and to minimize dropout between the initial test and the retest. A current illness question (In the past 30 days, have you been sick with a cold- or flu-like symptoms?) was included to identify and exclude participants who were acutely ill when evaluating the test-retest reliability of RSES scores.

#### Sample Size Determination

A sample size of 600 (n=200 per group) was needed to generate reliable estimates (ie, approximately 20 participants per parameter [15,16]) in the graded response model (GRM) [17]. For the test-retest reliability assessment, a sample of 100 participants provided 80% power to detect a significant difference between an intraclass correlation (ICC) of 0.80 and 0.69 (*F* test, α=.05, PASS 2020; NCSS).

### Participants

Smokers, switchers, and former smokers were recruited from varied locations around the United States from opt-in consumer research panels maintained by Kantar Profiles. Individuals sign up for such panels, agreeing to be invited to studies as they come up and to be compensated according to the panel’s practices. Soft target quotas were used to ensure a diverse sample of participants.

The participant inclusion criteria were as follows: must be >31 years of age (to allow for 10 years of smoking as an adult), reside in the United States, and have access to a device with internet access to complete the survey. The exclusion criteria were as follows: have a first-degree relative who is a current or former employee of the tobacco or e-cigarette industry, have a household member in litigation with a tobacco or e-cigarette company, and have participated in marketing research pertaining to tobacco or e-cigarettes in the past month.

### Ethics Approval

The study was approved by the Sterling institutional review board (protocol 8613, approved January 7, 2021). All participants signed a web-based informed consent document that accurately described the study procedures and their risks and benefits. Participants in the initial qualitative testing were compensated with US $65. Participants in the quantitative phase were compensated with “panel points” redeemable for goods in accordance with the standards of the research panel they had signed up for; these usually vary with the length of the survey. For the initial survey, this varied between US $1.00 and $4.50 for different panels. For the retest assessment, this varied from US $0.80 to $4.00. Study data were provided to the sponsor in a deidentified form and held securely and confidentially.

### Analytic Plan

A GRM [17] was fit in Mplus using the weighted least-squares estimator and θ parameterization. The GRM’s assumption of the ordinality of response categories was empirically evaluated by fitting a generalized partial credit model [18,19]. To evaluate the GRM’s assumption of unidimensionality, a Monte Carlo simulation (parallel analysis) with 10,000 randomly generated data sets was conducted using a principal components analysis extraction method. Test information and reliability were evaluated within the context of the GRM. The stability of the RSES was evaluated by calculating an ICC between RSES scores from the time 1 and time 2 surveys. Convergent validity was evaluated by calculating a Spearman correlation between RSES scores and self-reported global health status and by evaluating RSES scores by diagnostic status via independent-samples *t* tests.

As an indirect indication of the RSES’s ability to detect change, differences between smokers’ and former smokers’ scores were evaluated via an independent-samples *t* test. Similarly, using independent-samples *t* tests, exploratory analyses evaluated differences between switchers’ RSES scores and those of smokers and former smokers. To estimate the minimally important difference, the difference in adjusted (least-squares) mean RSES scores between those with and without respiratory symptom–relevant diagnoses was evaluated using a linear regression model controlling for tobacco use status (ie, study group membership). Finally, a receiver operating characteristic (ROC) curve was generated to determine the optimal RSES score cutoff in predicting self-reported respiratory symptom–relevant diagnosis.

## Results

### Participants

In total, 55,572 panelists were invited to participate. Of the 14,158 who started the recruitment screener, 1907 passed. The first 610 of these were enrolled in the study. All 610 participants started and completed the survey and comprise the analytic sample of 202 smokers, 208 switchers, and 200 former smokers.

Participant characteristics are presented in Table 1. Approximately half (285/610, 46.7%) of participants reported 1 or more diagnoses; 17% (104/610) reported a diagnosis of COPD. Per study requirements, all participants reported having smoked for at least 10 years, with a mean smoking history of 32.9 (SD 14.4) years. On average, smokers reported having smoked for 41.9 (SD 11.7) years, whereas switchers and former smokers smoked for approximately 28 years (mean 28.8, SD 13.8 years and mean 28.0, SD 13.2 years, respectively). Not surprisingly, switchers were on average younger than smokers and former smokers (mean 54.6 years vs mean 65.0 and 67.6 years, respectively), as the use of ENDS is more common in younger cohorts [20,21]. Nearly all smokers reported daily use of cigarettes (188/202, 93.1%), and switchers reported daily use of ENDS (193/208, 92.8%), respectively. Most switchers reported having used ENDS for more than 1 year (187/208, 89.9%). Switchers were using a variety of ENDS brands.

In total, 145 participants who completed the time 1 survey completed the time 2 survey. Of these, 128 participants who reported not being sick with a cold- or flu-like symptoms at either time point comprised the retest sample.

**Table 1:** Participant sociodemographic characteristics (N=610).^a^

Variable		Overall, n (%)	Smokers (n=202), n (%)	Switchers (n=208), n (%)	Former smokers (n=200), n (%)
**Gender**					
	Female	365 (59.8)	139 (68.8)	118 (56.7)	108 (54.0)
	Male	244 (40.0)	62 (30.7)	90 (43.3)	92 (46.0)
	Other	1 (0.2)	1 (0.5)	0 (0.0)	0 (0.0)
**Race**					
	White or Caucasian	569 (93.3)	190 (94.1)	190 (91.3)	189 (94.5)
	Black or African American	31 (5.1)	10 (5.0)	11 (5.3)	10 (5.0)
	Asian	7 (1.1)	1 (0.5)	5 (2.4)	1 (0.5)
	Native Hawaiian or Pacific Islander	3 (0.5)	0 (0.0)	2 (1.0)	1 (0.5)
	American Indian or Alaskan Native	17 (2.8)	5 (2.5)	8 (3.8)	4 (2.0)
	Other	8 (1.3)	1 (0.5)	3 (1.4)	4 (2.0)
**Ethnicity**					
	Hispanic	13 (2.1)	2 (1.0)	8 (3.8)	3 (1.5)
**Education**					
	Less than high school to high school graduate	175 (28.7)	66 (32.7)	53 (25.5)	56 (28.0)
	Some college or above	435 (71.3)	136 (67.3)	155 (74.5)	144 (72.0)
**Diagnostic status**					
	Allergies	173 (28.4)	62 (30.7)	56 (26.9)	55 (27.5)
	Asthma	39 (6.4)	11 (5.4)	11 (5.3)	17 (8.5)
	COPD^b^, emphysema, or chronic bronchitis	104 (17.0)	43 (21.3)	31 (14.9)	30 (15.0)
	Obesity	80 (13.1)	18 (8.9)	25 (12.0)	37 (18.5)
	Congestive heart failure	16 (2.6)	3 (1.5)	4 (1.9)	9 (4.5)
**Age (years)**					
	Minimum, Maximum	31, 88	32, 85	31, 82	38, 88
	Mean (SD)	62.3 (12.4)	65.0 (9.4)	54.6 (13.7)	67.6 (9.5)

^a^Participant sociodemographic characteristics and self-reported diagnostic status. Participants could endorse multiple races and diagnoses.

^b^COPD: chronic obstructive pulmonary disease.

### Rating Scale Functioning

Observed category averages and category thresholds derived from the generalized partial credit model were ordered as expected, providing empirical evidence that it requires a higher frequency of respiratory symptoms to endorse a more severe response option (eg, “Every day” vs “Most days”).

### Internal Structure

Results from the parallel analysis revealed a single significant factor (eigenvalue=3.14), providing support for unidimensionality of the RSES. This factor accounted for 62.7% of the variance.

### Graded Response Model

Fit statistics for the initial GRM indicated a poor fit of the data to the model (χ^2^_5_=285.086, *P*<.001; comparative fit index=0.963; root mean square error of approximation=0.303; standardized root mean square residual=0.085). Because of the conceptual similarity between items, in conjunction with large observed modification indices, the model was rerun allowing for 2 correlated errors (“Morning Cough” and “Cough Frequently” and “Shortness of Breath” and “Easily Winded”). This model exhibited an acceptable fit (χ^2^_3_=3.056, *P*=.38; comparative fit index=1.000; root mean square error of approximation=0.006; standardized root mean square residual=0.005).

The RSES items’ discrimination parameters were approximately 1 or higher, suggesting that the items were effectively differentiating between respondents with different levels of respiratory symptoms. See Table 2 for the RSES items’ discrimination and difficulty parameters.

**Table 2:** RSES^a^ parameter estimates from the graded response model (GRM).^b^

Item	Item discrimination (SE)	Difficulty parameters			
		1 (Never vs rarely or higher) (SE)	2 (Rarely vs occasionally or higher) (SE)	3 (Occasionally vs most days or higher) (SE)	4 (Most days vs every day) (SE)
1	0.714 (0.067)	−0.334 (0.093)	1.010 (0.116)	2.083 (0.187)	3.385 (0.308)
2	0.971 (0.078)	−0.183 (0.075)	1.032 (0.094)	1.953 (0.146)	2.866 (0.225)
3	1.121 (0.102)	0.127 (0.068)	0.921 (0.085)	1.599 (0.120)	2.410 (0.181)
4	1.110 (0.099)	−0.055 (0.069)	0.857 (0.084)	1.595 (0.118)	2.421 (0.176)
5	3.179 (0.885)	0.337 (0.053)	1.113 (0.072)	1.767 (0.107)	2.282 (0.151)

^a^RSES: Respiratory Symptom Experience Scale.

^b^This table shows the parameter estimates (item discrimination values and difficulties) generated from the GRM. See Textbox 1 for item content.

### Test Information and Reliability

**Figure 1:**
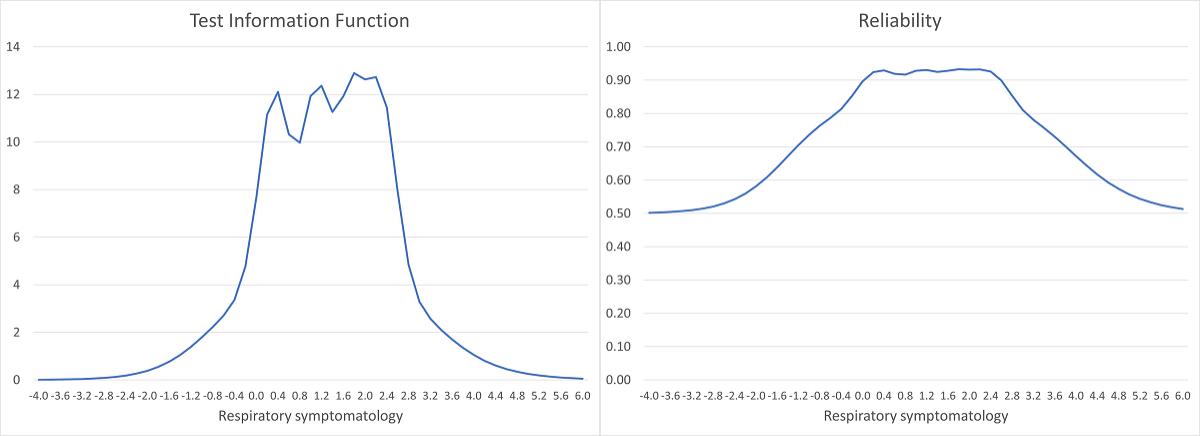
Test information function (TIF, left) and reliability generated from the graded response model (GRM). This figure depicts the amount of information (precision) that the Respiratory Symptom Experience Scale (RSES) provides across different levels of respiratory symptoms (θ, with mean 0, SD 1). The reliability function (right), derived from the TIF, illustrates how reliability within the context of the GRM varies across different levels of the latent trait. The RSES exhibits the highest reliability (reliability of 0.80 or higher) from θ of −0.40 to 3.00.

The test information function produced from the GRM (Figure 1) indicates that the RSES most precisely estimates respiratory symptoms from θ of 0.2 to 2.4. The reliability function (derived from the test information function by dividing information by information + 1) [22] indicates that the RSES exhibits a reliability of 0.80 or higher from θ of −0.40 to 3.00.

Given the high correlation between the GRM-derived scoring and the raw scores (*r*=0.94) and the complexities associated with using scoring from a 2-parameter item response theory model, it is recommended that researchers use the raw RSES item scores to calculate a composite (mean). Therefore, raw scores were used for the remaining analyses.

### Test-Retest Reliability

Test-retest reliability was good (absolute ICC=0.89).

### Differences Between Smokers and Former Smokers

Smokers’ RSES scores were significantly higher than Former Smokers’ scores (*t*_400_=3.87, *P*<.001; *d*=0.39, a small to medium effect size [23]), providing initial support for the RSES’s ability to detect change over time (Table 3). Given the observed differences in years smoked between study groups, these analyses were replicated using linear regression controlling for years smoked. The adjusted (least-squares) means were similar to the unadjusted means (smoker mean 2.00, former smoker mean 1.79), and the groups’ RSES scores remained significantly different (*P*=.02). Sensitivity testing using the nonparametric Mann-Whitney *U* test yielded the same statistical conclusion.

**Table 3:** Mean RSES^a^ score by the study group.

Study group	RSES score^b^, mean (SE)
Smokers	2.09 (0.06)
Former smokers	1.75 (0.06)
Switchers	1.72 (0.05)

^a^RSES: Respiratory Symptom Experience Scale.

^b^Mean RSES scores for the 3 study groups. Smokers’ scores significantly differed from Former smokers’ scores and from switchers’ scores. Former smokers’ and switchers’ RSES scores did not significantly differ.

### Differences Between Switchers and Smokers and Former Smokers

Switchers’ RSES scores were significantly lower than smokers’ (*t*_374.84_=4.59, *P*<.001; *d*=0.46, a small to medium effect size [23]; Table 3) and did not differ from former smokers’ (*t*_382.44_=0.34, *P*=.74; *d*=0.03). These conclusions remained when regression models controlled for the number of years smoked: adjusted (least-squares) means were similar to the unadjusted means (switcher mean 1.76), and the RSES scores of smokers and switchers (*P*=.005) remained significantly different, whereas the scores of switchers and former smokers did not differ (*P*=.68).

### Convergent Validity

As anticipated, higher RSES scores were related to poorer self-reported health status (*r_s_*=0.38, *P*<.001). With respect to known-groups validity, RSES scores were significantly higher among participants who reported 1 or more respiratory symptom–relevant diagnoses (mean 2.16, SD 0.92) compared with those who did not (mean 1.58, SD 0.64; *t*_496.07_=8.86, *P*<.001, *d*=0.74, a medium to large effect size). RSES scores were also significantly higher among participants with COPD (mean 2.76, SD 0.92) than those without COPD (mean 1.67, SD 0.68; *t*_126.94_=11.55, *P*<.001, *d*=1.52, large effect size). Nonparametric testing (Mann-Whitney *U* test) yielded the same conclusions.

### Association Between RSES and COPD

In linear regression with self-reported COPD and non-COPD diagnoses (ie, asthma, allergies, congestive heart failure, and obesity) as predictors, these diagnoses accounted for 29.2% of the variance in RSES scores. COPD diagnosis accounted for the majority of this variance (20.6%; *P*<.001) in models adjusting for non-COPD diagnoses. Controlling for age and years smoked did not have a material impact on results, with a small increase in the overall variance accounted for by the model (31.1%), and COPD diagnosis was still significantly related to RSES scores (*P*<.001) and explained 17.0% of the variance.

Finally, to directly quantify the relationship of RSES scores to self-reported COPD diagnosis, a logistic regression was run with COPD diagnosis as the outcome and RSES scores as univariate predictors. The analysis showed that with every 1 unit increase in mean RSES scores, the odds of COPD were 4.72 times greater (95% CI 3.47-6.40; *P*<.001).

### Minimally Important Difference

The difference in adjusted means for those with (least-squares mean 2.16, SE 0.05) and without (least-squares mean 1.58, SE 0.04) respiratory symptom–relevant diagnoses was 0.57 (*P*<.001). These results suggest that changes of about half a point or more in RSES scores reflect meaningful differences or changes in frequency of respiratory symptoms.

### Identifying the Optimal Cut-Point Using ROC

The ROC analysis yielded an area under the curve of 0.69 and identified a score of 2.0 as the optimal cut-point for identifying diagnosed individuals (specificity=0.76, sensitivity=0.57).

### Administration and Scoring

The RSES was developed and validated in electronic form, and electronic administration is recommended. RSES instructions are presented on a screen before item 1 (Textbox 1). To facilitate administration on a small-screen device, each RSES item is administered on a separate screen with the following instructions on each screen to remind participants of the recall period: “Over the past 30 days, how often did you experience the following?” RSES items are administered in a fixed order (Textbox 1), and the response option order is also fixed. Given the high correlation between the GRM-derived scoring and the raw scores observed here (*r*=0.94) and the complexities associated with using scoring from a 2-parameter item response theory model, it is recommended that researchers use the raw RSES item scores to calculate a composite. A composite score is calculated by taking the average of the 5 RSES items. If an item is missing, a composite should not be calculated.

## Discussion

### Summary and Strengths

The RSES fills an important gap in the existing toolkit of respiratory symptom patient-reported outcome measures, as it is a validated questionnaire appropriate for use with adult current and former smokers without a formal diagnosis of COPD.

The RSES is likely to be useful in many contexts, including assessing the health consequences of tobacco product use and the relatively immediate health benefits of quitting smoking. Demonstrating the improvement of respiratory symptomology associated with quitting smoking has implications for public health, as the frequency of respiratory symptoms has been shown to be associated with increased motivation to quit smoking [24-27]. Accordingly, ameliorating respiratory symptoms may be a particularly motivating driver for smokers to stop smoking, as respiratory symptoms may be experienced as tangible and relatable health effects that reflect more immediate consequences of quitting smoking.

RSES development and validation was a rigorous multiphase process conducted in accordance with guidance and best practices. The RSES is understood and correctly interpreted by diverse samples of respondents with respect to age, ethnicity, and health literacy status. Results from the psychometric evaluation provide evidence of unidimensionality, high levels of measurement precision across a range of relevant symptom levels, convergent validity, and stability over a 1-week interval.

Although this study’s cross-sectional design did not permit evaluation of intraindividual change in respiratory symptoms with smoking abstention, observed differences between smokers’ and former smokers’ RSES scores suggest that the RSES may be sensitive to changes that occur when a smoker stops smoking. Former smokers who switched to ENDS reported significantly lower respiratory symptoms than current smokers, even after accounting for years of smoking. Moreover, switchers’ level of self-reported respiratory symptoms did not significantly differ from former smokers who no longer using any tobacco products. These results suggest that switching from cigarettes to ENDS may improve smokers’ respiratory symptoms.

Our study included smokers who switched completely to ENDS, finding that their level of respiratory symptoms differed from that of smokers and was close to that of former smokers who were not using ENDS. The scale would also likely be useful for testing respiratory symptoms in smokers who have switched to other noncombusted sources of nicotine, including heated tobacco products [28], which involve inhalation of an aerosol derived from heating—but not burning—tobacco. It would also be of interest to assess respiratory symptoms in smokers who have not switched completely away from cigarette smoking but are engaged in dual use of both cigarettes and ENDS (or heated tobacco products). It is likely that in such dual-user populations, the degree of recovery from smoking-related respiratory symptoms may depend on how much they are still smoking. Observational studies (eg, [29]) suggest that dual users often substantially reduce their cigarette consumption, which could result in reduced respiratory symptoms, though likely to a lesser degree than switching completely away from smoking. Future studies might also fruitfully include a group of never smokers as a reference point for comparison with groups with a history of smoking.

The RSES significantly differentiated those with and without relevant respiratory conditions, even after controlling for smoking status, and an average score of 2.0 (at least monthly experience of symptoms) can help identify individuals with meaningful symptoms.

COPD diagnosis was significantly related to RSES scores above and beyond other respiratory symptom–related diagnoses, suggesting that the symptoms assessed by the RSES may have some specificity for COPD. Further, each 1 unit increase in RSES scores was associated with nearly 5 times greater odds of COPD. Although the RSES is not intended to function as a screener or diagnostic tool for COPD, results from these analyses suggest that respiratory symptoms assessed by the RSES may be related to eventual pulmonary disease.

The RSES was developed in a way that facilitates electronic administration on devices of different size (eg, a single item was administered on each screen to prevent the need for scrolling, the item stem was administered on each screen along with the item to remind participants of the timeframe of reference, etc). This feature may be useful, as self-report questionnaires may be increasingly administered across various electronic platforms.

### Limitations

Study groups differed in age and years smoked. Although this finding is not particularly surprising (eg, switchers were expected to be younger than smokers and former smokers), statistically controlling for these variables may not completely account for intergroup differences, and there could be other unmeasured differences not included in the analysis.

As this study was not longitudinal, it was not possible to draw causal inferences of the relationships between changes in tobacco use and a subsequent change in respiratory symptoms. Future longitudinal research should evaluate an intraindividual change in smokers’ respiratory symptoms, as assessed by the RSES, after stopping smoking and complete switching to ENDS.

The study relied exclusively on self-report; tobacco use was not biochemically verified, and diagnostic status was self-reported. Additionally, the survey was fielded during the COVID-19 pandemic, so it is possible that COVID-related symptoms added unaccounted variation in symptom reporting.

### Conclusions

Research evaluating respiratory symptoms among current and former smokers without diagnosed clinical pulmonary disease has been hindered due to the lack of a self-report questionnaire valid for assessing respiratory symptoms among nondiseased populations. The RSES fills an important gap in the existing toolkit of respiratory symptom patient-reported outcome measures as it is psychometrically validated and appropriate for use among adult current and former smokers without a formal diagnosis of COPD.

## Abbreviations

COPD: chronic obstructive pulmonary disease
ENDS: electronic nicotine delivery systems
GRM: graded response model
ICC: intraclass correlation
ROC: receiver operating characteristic
RSES: Respiratory Symptom Experience Scale
